# Changing diets and the transformation of the global food system

**DOI:** 10.1111/nyas.14446

**Published:** 2020-07-26

**Authors:** Sonja J. Vermeulen, Toby Park, Colin K. Khoury, Christophe Béné

**Affiliations:** ^1^ CGIAR System Organization Montpellier France; ^2^ Hoffmann Centre for Sustainable Resource Economy London UK; ^3^ Behavioural Insights Team London UK; ^4^ International Center for Tropical Agriculture (CIAT) Palmira Valle del Cauca Colombia

**Keywords:** diets, behavioral change, sustainability, health, policy

## Abstract

An aspirational global food system is one that delivers across a suite of the Sustainable Development Goals (SDGs), including universal access to healthy diets, which can also codeliver on climate and environment SDGs. The literature has downplayed the relative contribution of dietary change to sustainable food systems. In this perspective article, we argue that the potential for positive transformational change in diets should not be underestimated, for two sets of reasons. First, the dynamism of diets over long‐term and, especially, recent history shows the potential for rapid and widespread change, including toward more diverse and healthier diets. Second, contemporary behavioral research demonstrates promising tactics to influence consumers’ dietary choices. Since the entire food system creates the circumstances of those choices, the most effective strategies to shift diets will involve multiple approaches that deliberately aim not just to influence consumers themselves but also to incentivize all actors in the food systems, taking into account multiple agendas and values. The effectiveness of actions will depend on the political economy at local, national, and global levels. Overall, there are reasons to be hopeful about the potential for accelerated global dietary change, given both historic trends and the growing suite of tools and approaches available.

## Introduction

To realize the vision of the Sustainable Development Goals (SDGs) and, in particular, to achieve zero hunger (SDG2) while keeping climate change under 2 °C (SDG13), we will need not only greater food security among poor or marginalized people, but also a shift to greater sustainability in patterns of consumption among wealthy people (SDG12).^1^, ^2^ Modeling studies suggest that it is, in theory, possible for everyone to have a nutritious diet made up of diverse foods that could vary among cultures, without breaching the 2 °C limit, even with population growth to 2050.^3^, ^4^ But this would involve drastic changes in diets for many. For undernourished populations, it would involve diversifying the types of foods consumed, combined with a moderate increase in consumption of animal‐source foods, especially for children, while for people at the higher end of the consumption spectrum, it would instead involve decreasing energy intake and shifting toward a more plant‐centric diet, with a higher volume and diversity of pulses, nuts, whole grains, tubers, fruits, and vegetables.^5^, ^6^

Yet, diets are often considered relatively difficult to change from three related perspectives. The first is one of principle, that what we eat is a matter of sovereign personal or collective choice.^7^ The second is based on practical experience, for example, evidence of poor long‐term outcomes from dietary weight‐loss programs.^8^ The third is theoretical, based on the premise that there are few levers for society‐wide behavioral change. For example, a common view among the environmental community is that achieving emissions reductions via changes in agricultural practices and technologies will be much easier to accomplish than via dietary change among billions of consumers.^4^, ^6^

To date, governments, industry, and civil society organizations in high‐income countries have faced barriers to taking action on diets in a holistic manner, leading to modest and slow improvement.^5^, ^9^ In many low‐income countries, governments have implemented health and agricultural development programs to reduce undernutrition, and are beginning to take steps to address poor dietary quality, but have struggled to embrace more comprehensive food system strategies that would balance multiple desired outcomes related to nutrition, food security, climate, environment, and socioeconomic development.^10^

In this paper, we set out to challenge the view that changing food demand is more difficult than changing food supply, with a more hopeful vision for dietary transformation. We contend that diets are in constant flux, with large changes evident even within single generations and across very different cultures. Change in diet, indeed, is not only possible, but is the norm. In addition, behavioral research is generating a wide suite of tools and approaches to situate healthy and sustainable eating as the normal, easy, and appealing choice. Holistic approaches that change diets in tandem with food system‐wide transformation may meet with greater success, since consumer preferences are far from independent of the multitude of supply‐side drivers and political economy of food availability and access. We add to a suite of voices^5^, ^6^, ^11^ that contend that a broad approach to healthy and sustainable diets—one that involves new tools for behavioral change but also encompasses actions across whole food systems—will be essential to enabling the needed transformations at the global scale.

To address those issues, we conducted a thorough review of the literature on dietary change in relation to food and land system change. We started with the major global foresight and analytic studies published since 2015. From these, we moved to explore some of the less well‐elaborated areas of enquiry among food system specialists (as opposed to nutrition, public health, and behavioral specialists), particularly the evidence on effective strategies to promote dietary change and the cross‐food‐system linkages that create the enabling environments to promote simultaneous transformation of diets and food systems. We sought out published sources related to both high and lower‐income countries, though on strategies for dietary change we found substantially more literature related to high‐income countries.

## Diets in flux

Diets can change very quickly, within a generation. A global nutrition transition, from diets with a high proportion of a limited set of staples toward more diversified diets that are higher in energy and macronutrients—as well as in specific food groups, such as meat, sugar, processed foods, and foods eaten outside the home—has been well documented.^12^, ^13^, ^14^, ^15^, ^16^

People, almost without exception, are eating more food than their grandparents did. The mean 2250 calories humanity consumed in 1960 rose to 2800 by 2010, a 24% increase globally in half a century. Similar trends are true for protein (+25%), fat (+46%), and food mass (+25%).^17^

Paradoxically, individual diets have diversified, while global diets grew more homogenous, with national food supplies becoming 36% more similar over the half century.^17^ Production and consumption of vegetable oil from oilseed crops, such as soybean, palm oil, sunflower, and rapeseed, has also risen, partly associated with the rise in intake of processed foods and snacks.^18^, ^19^ Intakes of vegetables, fruits, nuts and seeds, and polyunsaturated fatty acids have shown positive trends,^20^, ^21^ but still remain below recommended levels in most regions,^22^ particularly among poorer people, for whom recommended levels of fruits and vegetables are often simply not affordable.^23^, ^24^ Nonetheless, in some countries, such as South Korea and Japan, healthier eating patterns have prevailed.^25^, ^26^

Meat consumption increased globally by 20 kg per capita between 1961 and 2014, though high‐income regions, where consumption is the highest, experienced stagnation in total consumption and a decline in beef relative to chicken.^18^ Food intake levels are closely associated with wealth.^27^ However, more recent data suggest a rise in low‐meat diets across several European countries; supermarkets reported plant‐based products to be their biggest source of growth in 2018 and wider industry research revealed that products labeled as vegan increased sales by 276% in a year.^28^, ^29^, ^30^ However, these trends reflect only a small segment of the global population, rising from a low baseline of vegetarians and vegans. As yet, these retail trends have not made a significant dent in OECD livestock import and export figures.^31^ They also sit against a backdrop of far bigger global increases in meat consumption, with billions across Asia and Africa entering the middle class, driving an expected 74% increase in demand for meat by 2050.^32^

The drivers of rapid dietary change include urbanization, rising incomes, and societal changes, such as greater participation by women in labor markets, but also developments in technology, business strategies, and public policy.^11^, ^33^, ^34^, ^35^ In the second half of the 20th century, poverty‐reduction and economic development imperatives in low‐ and middle‐income countries focused on multiplier effects from investment in agriculture.^34^ In parallel, the scaling out of high‐yielding wheat and rice in Asia under the green revolution brought lower and more stable consumer prices.^36^ Brazil's 40‐fold increase in per capita intake of soy was brought about in large part due to intense investment in the domestic cultivation of the crop, supported by state economic incentives and agricultural research.^37^ In high‐income countries, post‐war food security policies prioritized maximizing macronutrient availability through supply‐side policy instruments, such as marketing boards, land taxes, and farm subsidies.^11^, ^38^

Beyond agricultural and food security policies, trade policy has been pivotal to dietary change. A key turning point was the Uruguay Round of the General Agreement on Tariffs and Trade (GATT) in 1994, which opened long‐protected domestic food markets through pledges to harmonize standards and to reduce both tariffs and nontariff barriers.^38^ Global trade frameworks under GATT and the World Trade Organization, and subsequent free trade agreements, such as the North American Free Trade Agreement, have been identified as catalysts for vertical and horizontal integrations in food supply chains, value addition via greater processing, and, in turn, increased availability and consumption of food,^39^, ^40^ particularly unhealthy processed foods high in sugar, salt, and added fats and low in fiber and other nutrients, leading to an upsurge in obesity in all regions.^38^, ^41^, ^42^ The middle segments of food supply chains—particularly processors—increasingly drove a gap between the nutritional content and prices of agricultural produce versus the foods available for consumers in retail and food service facilities.^43^, ^44^, ^45^, ^46^, ^47^ Highly processed foods comprise 75% of calories in the United States,^45^ and other countries are catching up; purchase of processed foods has recently grown at 50% per year in China.^48^

The outcomes of the rising availability and affordability of diverse foods have been mixed.^20^ The global rate of hunger declined from 19% to 12% between 1990 and 2012,^49^ although it has slightly risen again since 2015.^50^ Over the same period, the percentage of stunted children fell from 40% to 24%.^51^ However, this transition has also been associated with a global rise in obesity, heart disease, and type 2 diabetes.^9^, ^13^ The combined number of overweight and obese adults globally is projected to rise from 1.33 to 3.28 billion between 2005 and 2030.^51^, ^52^ In Nigeria and Ethiopia, the number of adults with diabetes is projected to double from 2011 to 2030.^53^ The transition toward more processed food may have contributed to reducing hunger by making calories, protein, and fat more widely available worldwide.^54^ But highly processed foods often have unhealthy nutritional profiles and fail to improve the sufficiency of vital micronutrients, such as vitamin A, iron, and zinc, in regions where deficiencies are significant in diets.^5^, ^15^, ^55^, ^56^

## Strategies to change consumers’ dietary choices

In theory, a widespread shift in diet is achievable: there are no major technological challenges to address, and there is little that would prevent individuals in high‐income countries from changing to diets that would offer benefits to both health and environment. Yet, unhealthy dietary habits remain prevalent. The bulk of studies to date show that it is possible to influence positive dietary change, but achieving changes in habit, tastes, culture, and norms presents challenges.^57^ This section examines some of the ways we might begin to encourage those changes. We focus on high‐income countries because of the concentration of research there, but scattered studies conducted in low‐income and middle‐income countries, whether large economies, such as China and India, or smaller, such as Malawi and Kenya, indicate similar behavioral patterns among consumers.^58^, ^59^

### Limits of conventional approaches

Historically, scholars have conceptualized behavioral changes predominantly through the lens of rational choice: that as consumers we carefully consider our options in order to make conscious decisions that maximize the utility, or benefit, to ourselves.^60^ Elements of this understanding of human behavior underpin, or are implicit within, the most familiar tools of policy makers and campaigners.^61^ Education, awareness raising, food labels, and other forms of information provision broadly aim to overcome information deficit or asymmetry, on the premise that rationally optimal decisions can only be made in possession of full knowledge.^62^ Likewise, attitudinal campaigns and incentives in retail environments may aim to alter consumers’ preferences, which are founded on a mix of factors, including convenience, culture, personal identity, quality, freshness, enjoyment, and, to a lesser extent, health (to date, environmental concern is rarely mentioned).^63^

Labeling and other forms of information provision can be effective under the right conditions—for instance, evidence from public health research shows that point‐of‐choice information, for example, product labels and prompts, tends to be more effective than generic awareness‐raising activities, though all forms of information provision are likely to be most effective among the health‐literate and can thus exacerbate health inequalities.^64^ Analogously, the literature suggests that sustainability messaging tends to be more persuasive to those already onboard with the message, and so can further polarize attitudes.^65^

Moreover, information provision, logically, can only be effective when information deficit is a limiting factor.^66^ This is often not the case when it comes to sustainable consumption: evidence of a widespread value–action gap reveals that we often act unsustainably, prioritizing self‐interests, such as cost, convenience, and enjoyment, despite awareness and holding ostensibly sincere proenvironment attitudes.^67^, ^68^, ^69^ This apparent hypocrisy is enabled by a tendency to rationalize our behavior through various cognitive tricks, including motivated inattention (not thinking about issues which alert us to our inconsistencies or evoke guilt), moral licensing (using previous good acts to justify the bad), and motivated reasoning (reasoning toward the desired, not logical, conclusion).^70^, ^71^ Therefore, while awareness‐raising can be important, particularly to build public support for policy change, it is rarely the most effective route to individual behavioral change.^72^, ^73^, ^74^, ^75^ Indeed, while there will always be some consumers who are motivated to act more sustainably and only lack the information, information provision as a means to encourage more sustainable food choices is a broadly ineffective strategy.^76^

Finally, even where awareness is present, and intentions to eat healthier and more sustainably are absolutely sincere, practical barriers, such as poor availability of options or inconvenience, still arise, as well as psychological barriers, such as procrastination, lack of willpower, ingrained habit, or forgetfulness.^73^, ^77^ Biased or automatic processes of decision making may also create barriers to change, as they tend to err toward the familiar and the status quo^78^ and toward options that are perceived as socially normative.^79^

### Alternative strategies beyond information and awareness

Fortunately, many other strategies are available. A behaviorally informed approach must recognize that, contrary to conventional rational choice models, food choices are a product of both rational and automatic cognitive processes and are constrained by the physical, socioeconomic, and cultural structure of the food choice environment.^63^, ^80^ That is, in addition to individual preferences and perceived costs and benefits, consumption habits are profoundly influenced by the prevalence, layout, cost, and salience of options, by biased and nonconscious decision making, and by sociocultural norms and practices.^80^ It, therefore, stands to reason that rather than trying to change people's conscious lifestyle choices (through greater awareness or environmental concern), it may be more effective to edit various aspects of their choice environment: the options they are presented with, the sociocultural associations or perceived normality of those options, and the choice architecture (e.g., positioning, ordering, and context) within which they are offered.^63^

We identify three major themes of promising intervention. First, healthy and sustainable food must be made more *appealing*. Second, there is a direct relationship between the level of motivation required to do something and the ease of doing it,^81^ and seemingly trivial hassles can create disproportionate barriers.^82^ Healthy and sustainable food consumption must, therefore, be made *easy*, that is, available, convenient, prevalent, and, where possible, the automatic or default choice. Third, it must be perceived as *normal*, that is, familiar and socially normative, aligned with mainstream social identities rather than with counterculture niches. We expand on each strategy below.

#### Make it appealing

When people in higher‐income countries are asked why they eat what they eat, taste usually ranks top, followed closely by price, some way above concerns over health or the environment (the latter is rarely mentioned).^83^, ^84^ Indeed, while food marketed as healthy appeals to a niche market, it typically sells less than identical food marketed as delicious.^85^ For example, renaming “meat‐free” dishes to “field grown” and other names emphasizing decadence or experiential enjoyment significantly increased ordering rates.^86^ This work implies that vegetarian food often has connotations of being light, fresh, or healthy but also lacking or abstemious. Thus, in order to attract mainstream consumers, it would also have to provide options which are perceived as filling, hearty, and rich.^32^ This is in part an issue of framing and marketing,^86^ and in part one of developing new products.

Another way to make sustainable options more appealing is to make them cheaper relative to unsustainable options. Taxes and other price incentives can be highly effective, though levies or sin taxes may backfire because the payment provides a social license for the behavior,^87^ while payments or subsidies can crowd out intrinsic motivations to act virtuously.^88^ In this context, an example of an effective strategy may be the successful sugar tax in the UK,^89^ which incentivized manufacturers on reformulation, in order to avoid tax thresholds, rather than consumers to change their consumption habits. The same approach could use CO_2_ emissions per portion to incentivize innovative reformulation or development of sustainable food products.

#### Make it easy

Food choices are also shaped profoundly by habit and other factors beyond conscious awareness.^80^ One consequence of this reliance on automatic, intuitive, and heuristic decision making is a sensitivity to context and environmental factors.^90^ The strategies here are to make the healthy choice easier than the unhealthy choice (often highly processed convenience foods). For instance, increasing the number of available healthy and sustainable options, putting healthier and more sustainable options first on menus, moving their positioning in supermarkets (toward more salient places, such as end‐of‐aisle and eye‐height), and altering portion size are all effective techniques.^91^, ^92^, ^93^ These techniques predominantly target nonconscious processes, but where choices are more deliberate, ease is still important.^81^, ^82^ It is, therefore, important to overcome known barriers and frictions and to provide easy substitute products, which do not require deviation from the familiar. In Western culture, leading examples are burgers that blend meat and vegetables, or nonmeat burgers that mimic the appearance and mouthfeel of meat. While ease is often associated with unhealthy processed foods, there are opportunities both to increase the healthiness of processed options and to increase the ease of non‐processed options.

#### Make it normal

The social and cultural dimensions of food choices also deserve particular attention.^80^ As with any consumer choice, food consumption is partly an act of self‐expression of the norms or social expectations of the perceived in‐group. This may present a barrier to widespread adoption of plant‐based food, which, in the industrialized West, is often associated with a niche, minority identity,^94^ provoking a strong sense of otherness among meat‐eaters and associations of abstemiousness, weakness, or femininity.^93^ Overtly vegetarian branding or the segregation of products in supermarkets and menus tends to exacerbate this perception of otherness. For instance, research shows that having vegetarian items in a separate box on menus can reduce ordering rates by 56%,^95^ and that having “veggie only” refrigerators reduced sales compared to integrating products.^96^

The normalization of healthy and sustainable foods is also critical for another reason: wider evidence on environmental behavior shows that our willingness to act sustainably depends heavily on our perception that others do their share.^97^, ^98^, ^99^ Highlighting the increasing normality of eating less meat has been shown to be effective.^100^ In many public goods contexts, communicating the positive social norm helps promote the prosocial behavior; for example, telling people that most other people recycle,^101^ or pay their taxes on time,^82^ or use less energy,^102^ have all proven effective at promoting those behaviors.

Understanding the various conscious and unconscious processes described above and both the psychosocial and situational factors at play gives a broader set of tools to draw upon. Ultimately, the biggest impact will likely come from combining these approaches, both motivating the consumer by raising awareness and making healthy foods more appealing, but also creating an enabling environment in which it is easy and normal to eat healthier and more sustainable food (Table 1). These efforts should reinforce each other, as increased awareness and consumer demand drive policy, industry, and cultural change, which further normalize and remove frictions to healthier and more sustainable eating.

**Table 1:** Examples of strategies that can foster consumers’ choice transformations

Make it appealing Develop products, and market healthy and sustainable options, as appealing and delicious, rather than on messages of health, sustainability, or abstemiousness. Overcome negative connotations of weakness, or lack of satiety, that are often attributed to healthy food options, and avoid terms like dairy‐free or meat‐free that simply highlight what is lacking from the meal. Exceptions apply if targeting niche markets. A carbon tax on certain food products (e.g., ready‐made meals) can drive reformulation if set at appropriate thresholds of CO_2_ emissions per portion, such that the producer's incentive is to avoid the tax to maintain market share. A conventional sin tax (financially penalizing consumers who choose to eat certain products) may also be effective, though less politically feasible.
Make it easy Make healthy and sustainable options the default choice at catered events, on trains and airplanes, or in school and hospital canteens. Increase the number of healthy and sustainable options in menus, canteens, and supermarkets. Make these options more salient by putting them at the end of aisles and allocating them more shelf space. Put healthy and sustainable options first in canteens and on menus. Help consumers familiarize themselves with new healthy and sustainable foods, and overcome lack of recipe repertoire, by providing recipe cards in supermarkets. Provide simple substitutions to high‐impact and high‐volume food items, such as minced beef. This maintains familiarity and overcomes the hassle of learning new recipes or significantly altering the weekly grocery trip. Give timely prompts and reminders, for instance, by promoting product substitutions at the point of check‐out during online grocery shopping.
Make it normal Avoid segregating healthy and sustainable products. For instance, display burgers with different ingredient mixes in the same supermarket cabinet, regardless of refrigeration needs. Challenge niche‐identity associations through marketing and branding. Highlight the social norm, such as the new normal of vegan and vegetarian diets across Europe.^103^

## Strategies to integrate dietary change into food system transformation

People are at the center of our food systems and thus influencing consumer behavior and the food environments in which those behaviors take place is a central route to dietary change (see above).^5^, ^104^ But consumer behavior is not an independent, exogenous, demand‐side driver of food systems; instead, we need to consider how consumer behaviors and other food system functions interact and influence one another. Consumer choices drive agriculture and the food industry, but these choices (or lack of choices) are also shaped by food supply chain innovations and shocks and constraints,^105^ from droughts, to infrastructure failures, to trade bans.^106^ Larger‐scale drivers range from the political‐economic, such as industry concentration and lobbying power, to the biophysical, such as land and soil degradation,^5^, ^35^ or even major environmental tipping points, such as, for example, the melting of the Himalayan glaciers.^107^ Deliberate actions in the public or private sector to change these aspects of food systems are seldom, if ever, designed to change diets—but perhaps could become part of the future portfolio of levers for dietary change. This section considers the key arenas for action, highlighting some of the less discussed areas among the growing literature on strategies to achieve dietary change.^5^, ^6^, ^11^, ^46^, ^104^, ^108^, ^109^, ^110^, ^111^, ^112^

### The key role of the supply chain

A meaningful shift toward healthy and sustainable diets compatible with the SDGs would require supply to change in tandem with demand, increasing access to (including affordability of) better foods to prompt consumer demand at the same time as responding to that demand.^3^, ^6^ In terms of basic supply from agriculture, the current situation is far from what would support healthy food and sustainability. While farming already produces some foods in excess of global needs for human nutrition at the macro level (cereals and meat),^113^ supply of fruits and vegetables is only 42% of need across low‐income countries and 72% of need across all countries.^114^ A global shift toward healthy and sustainable diets would involve a transformation of crop patterns; a recent analysis of land use to deliver a healthy diet to everyone estimated that land used to produce cereals, oil crops, and sugar crops would need to be reduced by 150, 105, and 30 million hectares, respectively, and the production of vegetables and fruits to increase by 170 million hectares. Moreover, combining sustainable intensification with good governance to prevent the expansion of agricultural lands would offer a route to provision of universal healthy diets within the planet's environmental capacity.^6^, ^112^

The highly processed foods that make up substantial proportions of current diets are predominantly unhealthy.^115^, ^116^ But, if used well, processing can increase longevity, palatability, and nutrient availability while providing consumers with the convenient, consistent, and affordable foods that they often prefer.^117^ The “third stage” of the nutrition transition, following the first stage of traditional foods and second stage of industrially produced unhealthy ultra‐processed foods, is likely to be industrialized but healthier processed foods. These products—such as plant‐based packaged soups and bars that are high in fiber and micronutrients—currently have only a small niche market, but this is expected to grow rapidly.^46^, ^118^

Technological innovation in food is a probable game‐changer for healthy and sustainable diets.^105^ The world has moved quickly from conjecture to highly visible and successful start‐up companies creating lab‐based meats, edible insect products, and algal feed sources. The cases of Danone's expansion into plant milks and Tyson's investment in alt‐meats signal a move from the periphery to the mainstream for alternatives to animal‐source foods.^119^, ^120^ Production costs, and hence consumer prices, of alternatives to ruminant meat are currently prohibitive for the mass market, but are falling very rapidly and have potential to become less expensive than real meat, so that consumers could be soon leveraged on cost to eat healthier and more sustainably.^121^

More generally, affordability of food is central to broad‐based dietary change.^122^ Price changes, mediated by taxes or subsidies, are shown to be effective at raising consumption of healthy foods like fruits and vegetables and at reducing consumption of unhealthy foods like sugar‐sweetened beverages.^123^, ^124^ Yet, retail and catering food environments still offer fewer choices to less wealthy consumers, and poor health outcomes associated with food prices are more pronounced in low‐income than in high‐income countries.^125^, ^126^, ^127^ Ideally, food prices should include the environmental and health costs of food production and consumption^6^; current low food prices distribute those costs to producers, to the general public, and to future generations.^128^ Obviously, there is a delicate balance to be found between economic benefits to consumers and producers, as all food producers are consumers too, and many of the poorest farmers are net buyers of food.^129^ If we are to achieve healthy and sustainable diets, securing better wages and subsidizing poorer consumers through various forms of social protection may be a better alternative than subsidizing production of staple crops.

### Wider changes, including women's empowerment

Socioeconomic and policy drivers beyond the food system may outweigh changes within the agrifood sector in driving dietary change.^35^ Greater participation of women in informal and formal employment sectors outside the home has created larger incomes for women as well as rising demand for convenience foods, which have led to mixed nutritional outcomes in different countries.^130^, ^131^, ^132^ Investing in women's health and education and in family planning may provide more long‐term opportunity for achieving global adoption of healthy and sustainable diets in line with the SDGs than reforms directed at agriculture, food processing, and food retail or service subsectors.^133^ For example, a statistical analysis of historical national successes in reducing malnutrition and improving diets indicated that the most significant factor has been so far women's education, even more important than household income.^134^

Similarly, improving access to voluntary family planning, increasing educational and employment opportunities for women and adolescent girls, and empowering women with more decision‐making power over their own lives have also proven successful in improving nutritional outcomes for mothers and children.^135^ Likewise, a critical cobenefit of improving women's access to family planning and health and education services from the perspective of climate change and planetary health is the deceleration in population growth.^136^ Roll‐out of voluntary family planning programs that enable women to avoid unwanted births has shown rapid and large reductions in fertility rates.^137^ Countries that achieve reductions in their fertility rates are also observed to reap a demographic dividend in terms of an increase in GDP and household budgets, as the ratio of earners to dependents is higher and public infrastructure and services are more able to meet the needs of a smaller population.^137^

### Improving policy coherence

Siloed public policies coupled with weak incentives among potential lobby groups have hampered progress toward the global diets compatible with the SDGs. For instance, food considerations are almost entirely absent from climate policy and climate action at the national level; only two Nationally Determined Contributions (NDCs) to the Paris Agreement mention diets, and only 16 mention nutrition (out of 195 surveyed; CCAFS/CGIAR unpublished data). Too often, mainstream industry, while willing in principle to sell healthy and sustainable foods, follows a pathway of securing market share via sales of lowest‐common‐denominator, highly processed foods made from refined cereals, sugar, plant oils, dairy, meat, and salt.^43^, ^84^, ^138^ Meanwhile, nongovernmental organizations have tended to polarize between those concerned with development and food security, seeking to increase agricultural income and food availability,^139^ and those who emphasize environmental conservation and see food production primarily as a source of environmental degradation.^140^

Yet, recently more integrated visions have emerged among governments, businesses, and civil society. Intersectoral discussions around the SDGs show potential to unite competing agendas.^141^ This can widen the space for much‐needed debates and better‐informed decisions on managing trade‐offs within food systems.^10^ Governments that have traditionally focused on a simple national food security goal of maximizing the national breadbasket, measured in tons or calories, are showing greater concern toward issues of overweight and obesity, spurred in part by the global rise of noncommunicable diseases.^46^, ^104^, ^124^ Some are also entering into conversations around the environmental impacts of diets.^108^, ^142^ Retooling of agricultural subsidies to create environmental and nutritional incentives is a growing topic of policy attention in economies with global impacts, including China and the European Union.^143^, ^144^ Altogether, a window of opportunity is now open to reassess whether action on diets is feasible from local to global levels.

### The rising influence of social movements

In principle, bottom‐up and top‐down approaches to dietary and food system transformations should be complementary. Citizen‐led social media and social movements can influence social norms and sow the seeds of widespread behavioral change, enabling the effectiveness of more top‐down policies, while policies that enable or encourage new behaviors assist changes in consumer habits and can support social movements. The role of social movements and social media in driving transformation may be limited by their typically ephemeral nature—spikes in interest that often dissipate as quickly as they had emerged—and a tendency not to penetrate beyond a niche of young, urban, middle‐class membership or audience.^145^ Yet, as the recent experience with plastic waste suggests, a short‐term spike among a sufficiently large group that listens and cares may be enough of a hook for more durable responses by governments and businesses.

While the power of celebrities and individual champions to vitalize action receives much attention (e.g., promotion of vegetarianism by Indian film‐star Amitabh Bachchan and cricket captain Virat Kohli), more broad‐based social movements and organizations have possibly more decisive impact. Civil society's roles in system transformation include representing and raising the voice of marginalized communities, holding businesses and governments accountable, demonstrating new ways of producing and consuming, developing resilient local economies, providing services to underserved and isolated communities, defending people's rights or the protection of nature, advocating for different priorities or politics, and promulgating new social norms.^146^, ^147^, ^148^ In Brazil, for example, social movements have driven the creation of innovative national dietary guidelines that go beyond nutritional metrics to recommend behaviors around home cooking, communal eating, and discerning attitudes to food advertising, in turn catalyzing stronger regulations on the food industry and healthier school meals.^149^, ^150^, ^151^, ^152^

Yet, the separation between civil society and business is blurring, noticeably in the food sector. It has long been difficult to categorize many farmers’ organizations as either civil society or business groups,^153^ and new online platforms for social organization are softening this distinction further. For example, empirical experience in Indonesia shows how food activists use their online platforms for both marketing and community organization, while *prosumer* movements in high‐income countries are getting consumers more closely involved in design and production of food and farming systems.^154^

Several scholars recognize the city‐region as a key level of governance to unlock food system transformation,^108^, ^111^, ^155^ and new collaborations between civil society and municipal government are effectively observed. For instance, C40 Cities, a global network of mayors of 96 cities that account for a quarter of global GDP, works with civil society organizations in four areas: food production (urban agriculture to supply fresh vegetables but also to mitigate urban heat island effects), food procurement (to improve the supply of meals in cafeterias, hospitals, schools, and prisons), food distribution (largely through municipal markets), and food waste (working with community groups to redistribute food or to use it for animal feed or composting).^156^ In China, a pilot Healthy Cities initiative, enabled by increasing willingness of citizens to speak out on health and environmental issues, is tackling dietary change as part of a holistic health approach that links sanitation, medical services, healthy eating, and environmental pollution,^157^ with several cities acting also on food waste and farm footprint.^158^

### Radical versus moderate versions of transformation

Achieving healthy and sustainable diets requires, and is essential to, transformations of food systems, but different scholars have contrasting views of the changes in governance and power relations that such transformations might entail.^10^, ^159^ Some authors emphasize the ability of the private sector to innovate and adapt, and focus their recommendations on mechanisms such as financial or fiscal incentives to reorient the marketing strategies, business models, product formulation, and research and development of industry toward those transformations.^32^, ^43^, ^46^, ^84^ For others, transformation is not possible without addressing deep structural inequities.^33^, ^160^, ^161^, ^162^, ^163^ As Holt‐Giménez expresses, the food system is “structurally designed for profit rather than need, speculation rather than equity, and extraction rather than resilience.”^163^ For these scholars, food system transformation requires nothing short of dismantling capitalism. Other authors take a middle ground, in which disruption and significant changes are said to be possible without necessarily overthrowing the global economic regime, but not without addressing issues of political economy at multiple levels.^6^, ^11^, ^108^, ^155^, ^164^

Visions of a post‐transformed sustainable food system also differ tremendously. People hold values that determine the—sometimes very different—weight that they give to different issues, such as animal welfare, wilderness, workers’ rights, or reducing hunger.^165^ Yet, conversations across polarized positions are possible based on neutral analyses of how each side frames the issues and on efforts to find blended approaches or new configurations of the problem to create constructive dialogue.^159^, ^166^ Sustained engagement with multiple definitions of and approaches to sustainability and transformation may be key to practical progress,^10^ especially to ensure that the agendas of the most vulnerable producers and consumers are not marginalized.^167^

A start is being made: an emerging set of civil society initiatives, collective declarations, and public or private policy frameworks are opening the space for new conversations on diets and food systems. Examples include social movements on food across the Americas, Europe, Asia, and Africa,^146^, ^168^, ^169^ France's Circular Economy Roadmap (2018), the Nordic Council of Ministers’ solutions menu for food policy,^170^ the Food and Land Use Coalition,^171^ and the World Economic Forum's Shaping the Future of Food Initiative.^171^, ^172^ At the international level, the 10‐Year Framework of Programs on Sustainable Consumption and Production (2012–2022) is a global commitment to deliver on SDG12 by accelerating the shift toward sustainable consumption and production in countries across the income spectrum and is being implemented via the One Planet Network.

## Conclusion

Dietary change in the interests of human and planetary health cannot be considered too difficult to achieve when it has not yet been seriously attempted. While there are substantial countervailing forces—including highly successful business models that benefit from poor dietary choices, and governments not yet ready to consider diets a social issue rather than an individual one—there is also evidence that large‐scale transformation of diets may indeed be possible. In this perspective article, we have highlighted three strands of evidence that offer hope: the dynamic history of dietary change, growing insights from behavioral research, and the emergence of political spaces in which governments, social movements, and businesses across health and environmental sectors are able to open new conversations on what societies might demand of today's food systems.

## Competing interests

The authors declare no competing interests.
